# The genome sequence of the Dark Crimson Underwing moth, *Catocala sponsa *Linnaeus, 1767

**DOI:** 10.12688/wellcomeopenres.22759.1

**Published:** 2024-07-26

**Authors:** Gavin R. Broad, Natural History Museum Genome Acquisition Lab, Darwin Tree of Life Barcoding collective, Wellcome Sanger Institute Tree of Life Management, Samples and Laboratory team, Wellcome Sanger Institute Scientific Operations: Sequencing Operations, Wellcome Sanger Institute Tree of Life Core Informatics team, Tree of Life Core Informatics collective, Darwin Tree of Life Consortium

**Affiliations:** 1 Natural History Museum, London, England, UK

**Keywords:** Catocala sponsa, Dark Crimson Underwing, genome sequence, chromosomal, Lepidoptera

## Abstract

We present a genome assembly from an individual female *Catocala sponsa* (the Dark Crimson Underwing; Arthropoda; Insecta; Lepidoptera; Erebidae). The genome sequence spans 803.70 megabases. Most of the assembly is scaffolded into 32 chromosomal pseudomolecules, including the Z and W sex chromosomes. The mitochondrial genome has also been assembled and is 15.57 kilobases in length. Gene annotation of this assembly on Ensembl identified 13,493 protein-coding genes.

## Species taxonomy

Eukaryota; Opisthokonta; Metazoa; Eumetazoa; Bilateria; Protostomia; Ecdysozoa; Panarthropoda; Arthropoda; Mandibulata; Pancrustacea; Hexapoda; Insecta; Dicondylia; Pterygota; Neoptera; Endopterygota; Amphiesmenoptera; Lepidoptera; Glossata; Neolepidoptera; Heteroneura; Ditrysia; Obtectomera; Noctuoidea; Erebidae; Erebinae; *Catocala*; *Catocala sponsa* Linnaeus, 1767 (NCBI:txid753163).

## Background

*Catocala sponsa*, Dark Crimson Underwing, is a rather large, cryptically coloured moth, except when it shows its bright red hind wing. Previously considered a very localised speciality of the New Forest, with cycles of abundance and rarity ( South, 1907), this is a species which seems to be increasing in range and increasingly arriving in Britain as a migrant ( Randle *et al.*, 2019). In Kent, *C. sponsa* has been found breeding in oak woodland since 2019 ( Perry, no date). It was still a lovely surprise when GRB found one, a presumed immigrant, in his Kent garden. Despite its large size, care is needed when identifying *C. sponsa*, to differentiate it in particular from the similar *C. promissa* (Denis & Schiffermüller), Light Crimson Underwing. In *C. sponsa* the fore wing has a contrastingly paler patch against a more uniformly dark background and the hind wing has a sharply zigzagged black line within the red area (see Waring *et al.*, 2017).

The larvae of *C. sponsa* are wonderfully camouflaged as oak twigs, feeding on *Quercus robur*, Pedunculate Oak, from April to June ( Henwood *et al.*, 2020). They are specialised oak feeders, adapted to cope with tannins ( Roslin & Salminen, 2008). Adults are on the wing mainly in July and August in Britain and the eggs over-winter. Ranging widely across Europe and into Central Asia ( GBIF Secretariat, 2024), *C. sponsa* seems to be increasing in the northern edge of its range, such as in Britain and in Sweden (e.g., Franzén, 2004).

The species name ‘ *sponsa*’, from the Latin for ‘promised in marriage’ is one of a series of playful names which Linnaeus (1767) used for the red and blue ‘underwings’, which became the genus *Catocala*; Emmet ( Emmet, 1991) speculates on whether Linnaeus was referencing the flash of colour of otherwise hidden bridal underwear.

Here we present a chromosomally complete genome sequence for *Catocala sponsa*, based on one female specimen from Kent, England.

## Genome sequence report

**Figure 1.**
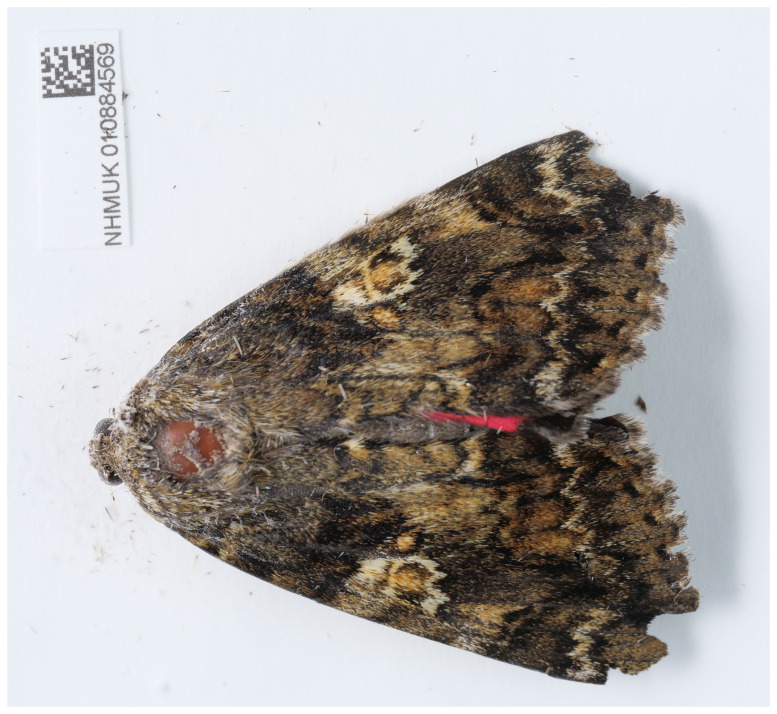
Photograph of the *Catocala sponsa* (ilCatSpon1) specimen used for genome sequencing.

The genome of an adult female *Catocala sponsa* ( Figure 1) was sequenced using Pacific Biosciences single-molecule HiFi long reads, generating a total of 61.86 Gb (gigabases) from 6.59 million reads, providing approximately 76-fold coverage. Primary assembly contigs were scaffolded with chromosome conformation Hi-C data, which produced 90.89 Gbp from 601.93 million reads, yielding an approximate coverage of 113-fold. Specimen and sequencing information is summarised in Table 1.

**Table 1.** Specimen and sequencing data for *Catocala sponsa*.

Project information			
**Study title**	Catocala sponsa		
**Umbrella BioProject**	PRJEB66398		
**Species**	*Catocala sponsa*		
**BioSample**	SAMEA112964387		
**NCBI taxonomy ID**	753163		
Specimen information			
**Technology**	**ToLID**	**BioSample** **accession**	**Organism part**
**PacBio long read sequencing**	ilCatSpon1	SAMEA112975590	abdomen
**Hi-C sequencing**	ilCatSpon1	SAMEA112975590	abdomen
**RNA sequencing**	ilCatSpon1	SAMEA112975590	abdomen
Sequencing information			
**Platform**	**Run accession**	**Read count**	**Base count (Gb)**
**Hi-C Illumina NovaSeq 6000**	ERR12102396	6.02e+08	90.89
**PacBio Revio**	ERR12085108	6.59e+06	61.86
**RNA Illumina NovaSeq X**	ERR12765150	6.13e+07	9.26

**Figure 2.**
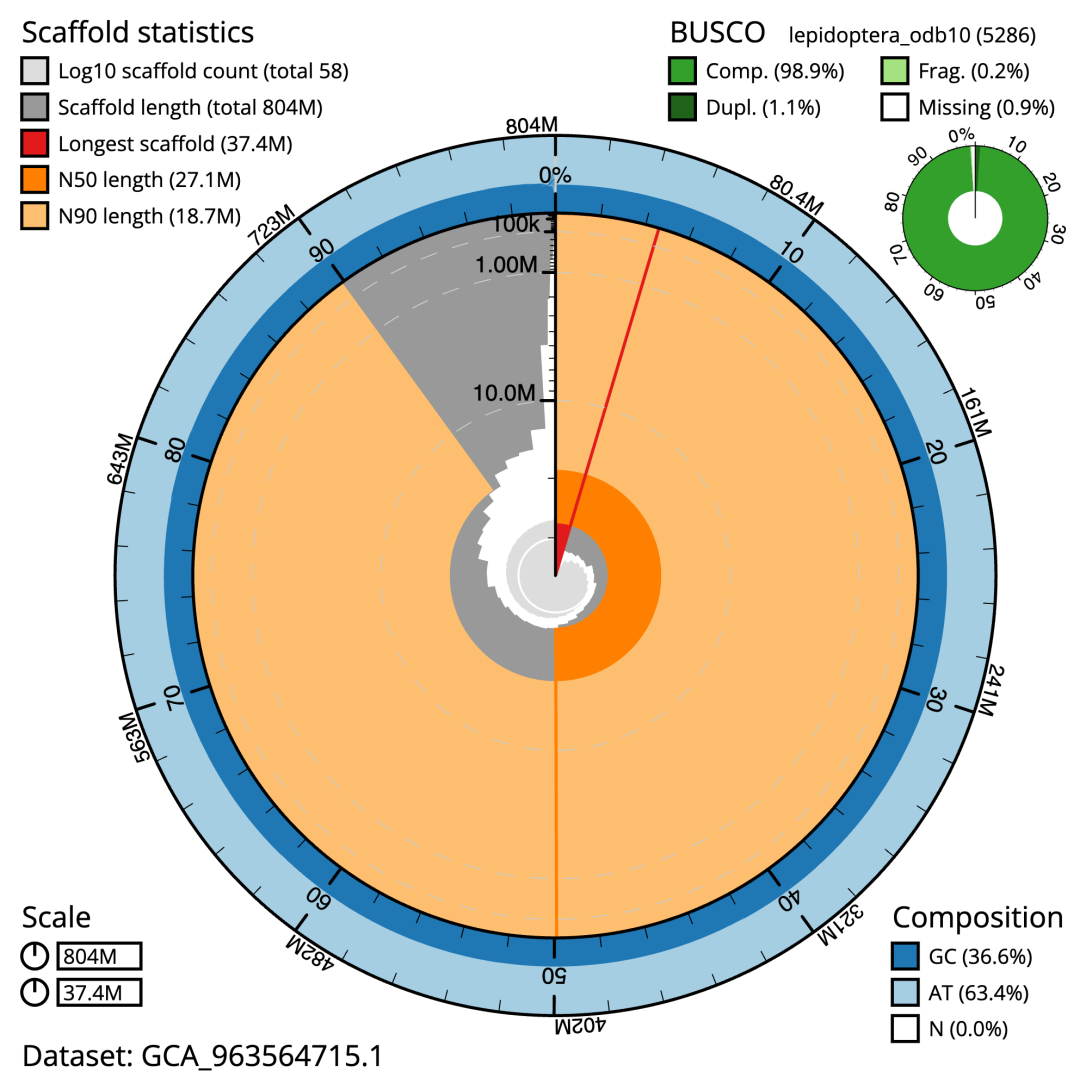
Genome assembly of *Catocala sponsa*, ilCatSpon1.1: metrics. The BlobToolKit snail plot shows N50 metrics and BUSCO gene completeness. $BTK_SNAIL_LEG An interactive version of this figure is available at https://blobtoolkit.genomehubs.org/view/Catocala_sponsa/dataset/GCA_963564715.1/snail.

**Figure 3.**
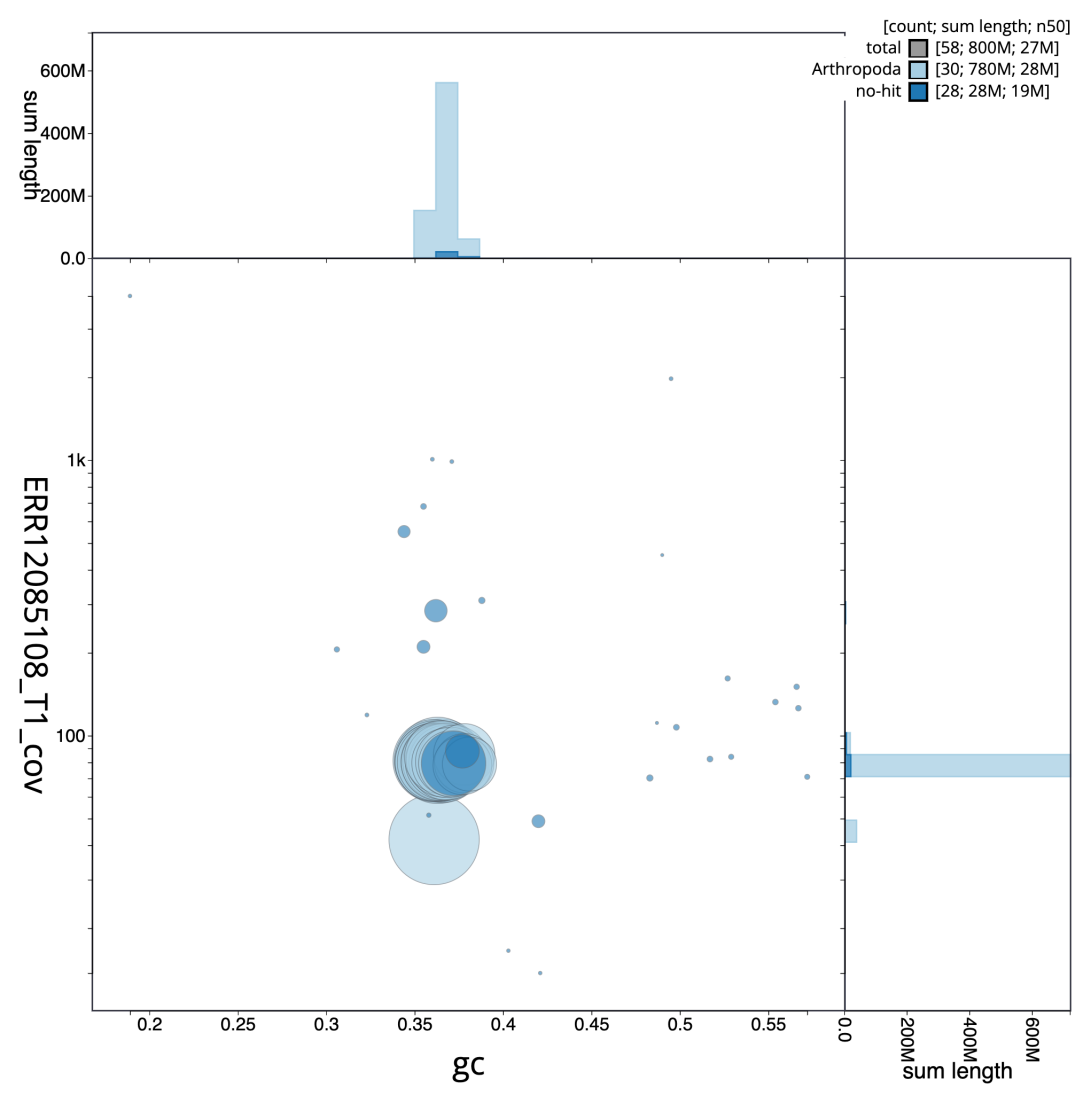
Genome assembly of *Catocala sponsa*, ilCatSpon1.1: BlobToolKit GC-coverage plot. Sequences are coloured by phylum. Circles are sized in proportion to sequence length. Histograms show the distribution of sequence length sum along each axis. An interactive version of this figure is available at https://blobtoolkit.genomehubs.org/view/Catocala_sponsa/dataset/GCA_963564715.1/blob.

**Figure 4.**
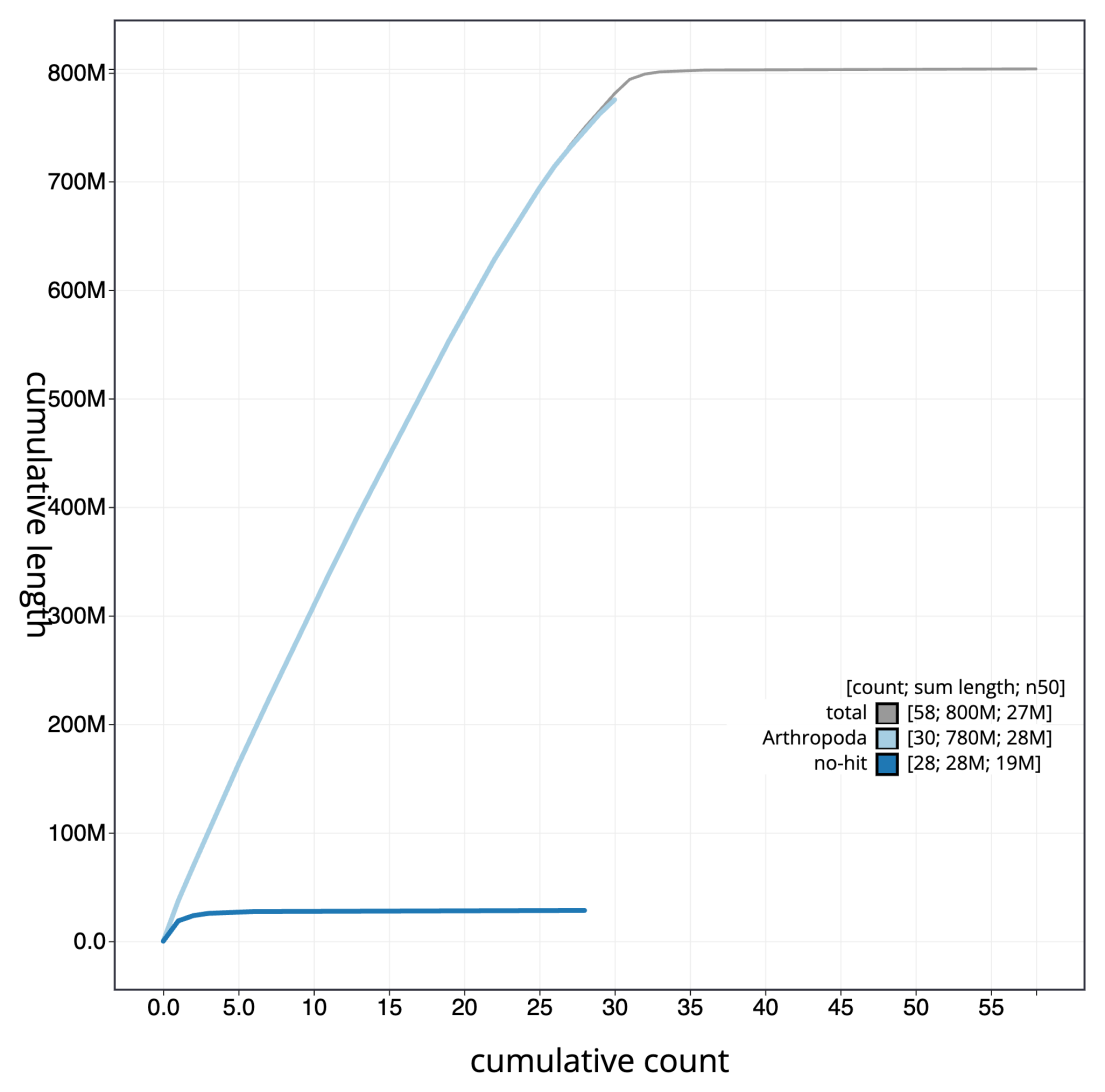
Genome assembly of *Catocala sponsa* ilCatSpon1.1: BlobToolKit cumulative sequence plot. The grey line shows cumulative length for all sequences. Coloured lines show cumulative lengths of sequences assigned to each phylum using the buscogenes taxrule. An interactive version of this figure is available at https://blobtoolkit.genomehubs.org/view/Catocala_sponsa/dataset/GCA_963564715.1/cumulative.

**Figure 5.**
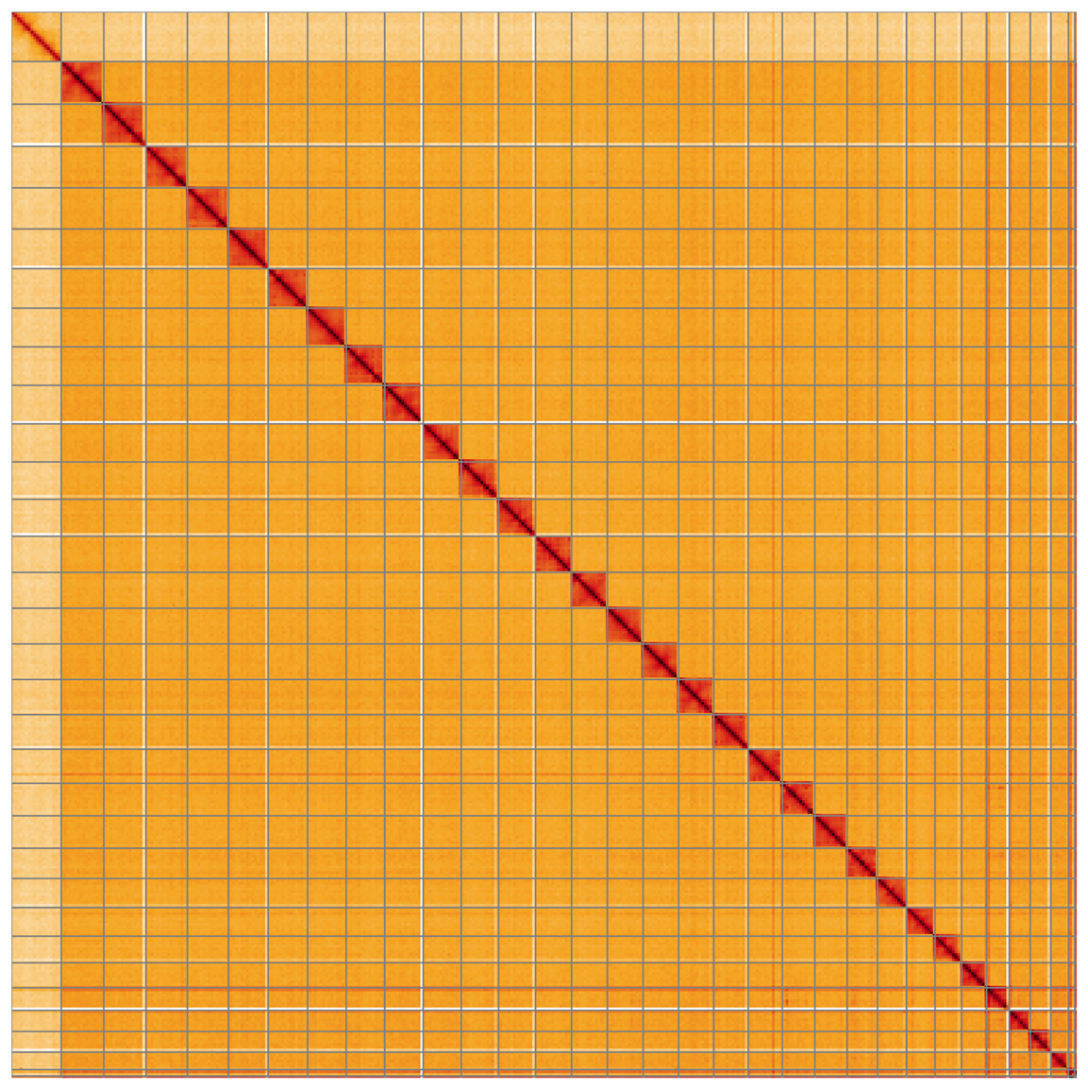
Genome assembly of *Catocala sponsa* ilCatSpon1.1: Hi-C contact map of the ilCatSpon1.1 assembly, visualised using HiGlass. Chromosomes are shown in order of size from left to right and top to bottom. An interactive version of this figure may be viewed at https://genome-note-higlass.tol.sanger.ac.uk/l/?d=LcOTwqcVQf6BFBvXAUEJ1Q.

Manual assembly curation corrected 7 missing joins or mis-joins, reducing the scaffold number by 3.33%. The final assembly has a total length of 803.70 Mb in 57 sequence scaffolds with a scaffold N50 of 27.1 Mb ( Table 2). The total count of gaps in the scaffolds is 64. The snail plot in Figure 2 provides a summary of the assembly statistics, while the distribution of assembly scaffolds on GC proportion and coverage is shown in Figure 3. The cumulative assembly plot in Figure 4 shows curves for subsets of scaffolds assigned to different phyla. Most (99.89%) of the assembly sequence was assigned to 32 chromosomal-level scaffolds, representing 30 autosomes and the Z and W sex chromosomes. Chromosome-scale scaffolds confirmed by the Hi-C data are named in order of size ( Figure 5; Table 3). Chromosome Z was identified by synteny to *Catocala fraxini* (GCA_930367265.1). Chromosome W was assigned by read coverage statistics. While not fully phased, the assembly deposited is of one haplotype. Contigs corresponding to the second haplotype have also been deposited. The mitochondrial genome was also assembled and can be found as a contig within the multifasta file of the genome submission.

**Table 2.** Genome assembly data for *Catocala sponsa*, ilCatSpon1.1.

Genome assembly		
Assembly name	ilCatSpon1.1	
Assembly accession	GCA_963564715.1	
*Accession of alternate haplotype*	*GCA_963565385.1*	
Span (Mb)	803.70	
Number of contigs	122	
Contig N50 length (Mb)	13.9	
Number of scaffolds	57	
Scaffold N50 length (Mb)	27.1	
Longest scaffold (Mb)	37.35	
Assembly metrics *		*Benchmark*
Consensus quality (QV)	66.7	*≥ 50*
*k*-mer completeness	100.0%	*≥ 95%*
BUSCO **	C:98.9%[S:97.8%,D:1.1%], F:0.2%,M:0.9%,n:5,286	*C ≥ 95%*
Percentage of assembly mapped to chromosomes	99.89%	*≥ 95%*
Sex chromosomes	ZW	*localised* *homologous pairs*
Organelles	Mitochondrial genome: 15.57 kb	*complete single* *alleles*
Genome annotation of assembly GCA_963564715.1 at Ensembl		
Number of protein-coding genes	13,493	
Number of non-coding genes	2,838	
Number of gene transcripts	25,901	

* Assembly metric benchmarks are adapted from column VGP-2020 of “Table 1: Proposed standards and metrics for defining genome assembly quality” from Rhie *et al.* (2021).

** BUSCO scores based on the lepidoptera_odb10 BUSCO set using version 5.4.3. C = complete [S = single copy, D = duplicated], F = fragmented, M = missing, n = number of orthologues in comparison. A full set of BUSCO scores is available at https://blobtoolkit.genomehubs.org/view/Catocala_sponsa/dataset/GCA_963564715.1/busco.

**Table 3.** Chromosomal pseudomolecules in the genome assembly of *Catocala sponsa*, ilCatSpon1.

INSDC accession	Name	Length (Mb)	GC%
OY751327.1	1	31.94	36.5
OY751328.1	2	31.67	36.5
OY751329.1	3	31.16	36.5
OY751330.1	4	30.89	36.0
OY751331.1	5	29.77	36.5
OY751332.1	6	29.62	36.5
OY751333.1	7	29.05	36.5
OY751334.1	8	28.95	36.0
OY751335.1	9	28.88	36.5
OY751336.1	10	28.61	36.5
OY751337.1	11	28.07	36.0
OY751338.1	12	27.72	36.0
OY751339.1	13	27.09	36.0
OY751340.1	14	26.91	36.5
OY751341.1	15	26.85	36.5
OY751342.1	16	26.66	36.5
OY751343.1	17	26.42	37.0
OY751344.1	18	25.91	36.5
OY751345.1	19	25.59	37.0
OY751346.1	20	24.42	36.5
OY751347.1	21	24.36	36.5
OY751348.1	22	22.68	37.0
OY751349.1	23	21.83	37.0
OY751350.1	24	21.53	37.0
OY751351.1	25	19.95	37.0
OY751352.1	26	18.75	37.0
OY751353.1	27	16.95	38.0
OY751354.1	28	15.88	38.0
OY751355.1	29	15.52	37.5
OY751356.1	30	13.05	38.0
OY751357.1	W	4.86	37.5
OY751326.1	Z	37.35	36.0
OY751358.1	MT	0.02	19.0

The estimated Quality Value (QV) of the final assembly is 66.7 with *k*-mer completeness of 100.0%, and the assembly has a BUSCO v5.4.3 completeness of 98.9% (single = 97.8%, duplicated = 1.1%), using the lepidoptera_odb10 reference set ( *n* = 5,286).

Metadata for specimens, BOLD barcode results, spectra estimates, sequencing runs, contaminants and pre-curation assembly statistics are given at https://links.tol.sanger.ac.uk/species/753163.

## Genome annotation report

The *Catocala sponsa* genome assembly (GCA_963564715.1) was annotated at the European Bioinformatics Institute (EBI) on Ensembl Rapid Release. The resulting annotation includes 25,901 transcribed mRNAs from 13,493 protein-coding and 2,838 non-coding genes ( Table 2; https://rapid.ensembl.org/Catocala_sponsa_GCA_963564715.1/Info/Index). The average transcript length is 21,228.05. There are 1.59 coding transcripts per gene and 7.19 exons per transcript.

## Methods

### Sample acquisition

An adult female *Catocala sponsa* (specimen ID NHMUK010884569, ToLID ilCatSpon1) was collected from Tonbridge, Kent, England, UK (latitude 51.19, longitude 0.29) on 2022-07-30, using actinic light. The specimen was collected and identified by Gavin Broad (Natural History Museum) and preserved by dry freezing at –80 °C.

The initial identification was verified by an additional DNA barcoding process according to the framework developed by Twyford *et al*. (2024). A small sample was dissected from the specimens and stored in ethanol, while the remaining parts of the specimen were shipped on dry ice to the Wellcome Sanger Institute (WSI). The tissue was lysed, the COI marker region was amplified by PCR, and amplicons were sequenced and compared to the BOLD database, confirming the species identification ( Crowley *et al.*, 2023). Following whole genome sequence generation, the relevant DNA barcode region is also used alongside the initial barcoding data for sample tracking at the WSI ( Twyford *et al.*, 2024). The standard operating procedures for Darwin Tree of Life barcoding have been deposited on protocols.io ( Beasley *et al.*, 2023).

### Nucleic acid extraction

The workflow for high molecular weight (HMW) DNA extraction at the Wellcome Sanger Institute (WSI) Tree of Life Core Laboratory includes a sequence of core procedures: sample preparation; sample homogenisation, DNA extraction, fragmentation, and clean-up. In sample preparation, the ilCatSpon1 sample was weighed and dissected on dry ice ( Jay *et al.*, 2023). Tissue from the abdomen was homogenised using a PowerMasher II tissue disruptor ( Denton *et al.*, 2023a). HMW DNA was extracted at the WSI Scientific Operations core using the Automated MagAttract v2 protocol ( Oatley *et al.*, 2023). The DNA was sheared into an average fragment size of 12–20 kb in a Megaruptor 3 system with speed setting 31 ( Bates *et al.*, 2023). Sheared DNA was purified by solid-phase reversible immobilisation ( Strickland *et al.*, 2023): in brief, the method employs a 1.8X ratio of AMPure PB beads to sample to eliminate shorter fragments and concentrate the DNA. The concentration of the sheared and purified DNA was assessed using a Nanodrop spectrophotometer and Qubit Fluorometer using the Qubit dsDNA High Sensitivity Assay kit. Fragment size distribution was evaluated by running the sample on the FemtoPulse system.

RNA was extracted from abdomen tissue of ilCatSpon1 in the Tree of Life Laboratory at the WSI using the RNA Extraction: Automated MagMax™ *mir*Vana protocol ( do Amaral *et al.*, 2023). The RNA concentration was assessed using a Nanodrop spectrophotometer and a Qubit Fluorometer using the Qubit RNA Broad-Range Assay kit. Analysis of the integrity of the RNA was done using the Agilent RNA 6000 Pico Kit and Eukaryotic Total RNA assay.

Protocols developed by the WSI Tree of Life laboratory are publicly available on protocols.io ( Denton *et al.*, 2023b).

### Sequencing

Pacific Biosciences HiFi circular consensus DNA sequencing libraries were constructed according to the manufacturers’ instructions. Poly(A) RNA-Seq libraries were constructed using the NEB Ultra II RNA Library Prep kit. DNA and RNA sequencing was performed by the Scientific Operations core at the WSI on Pacific Biosciences Revio (HiFi) and Illumina NovaSeq X (RNA-Seq) instruments. Hi-C data were also generated from abdomen tissue of ilCatSpon1 using the Arima-HiC v2 kit. The Hi-C sequencing was performed using paired-end sequencing with a read length of 150 bp on the Illumina NovaSeq 6000 instrument.

### Genome assembly, curation and evaluation

***Assembly***

The original assembly of HiFi reads was performed using Hifiasm ( Cheng *et al.*, 2021) with the --primary option. Haplotypic duplications were identified and removed with purge_dups ( Guan *et al.*, 2020). Hi-C reads are further mapped with bwa-mem2 ( Vasimuddin *et al.*, 2019) to the primary contigs, which are further scaffolded using the provided Hi-C data ( Rao *et al.*, 2014) in YaHS ( Zhou *et al.*, 2023) using the --break option. Scaffolded assemblies are evaluated using Gfastats ( Formenti *et al.*, 2022), BUSCO ( Manni *et al.*, 2021) and MERQURY.FK ( Rhie *et al.*, 2020).

The mitochondrial genome was assembled using MitoHiFi ( Uliano-Silva *et al.*, 2023), which runs MitoFinder ( Allio *et al.*, 2020) or MITOS ( Bernt *et al.*, 2013) and uses these annotations to select the final mitochondrial contig and to ensure the general quality of the sequence.

***Assembly curation.*** The assembly was decontaminated using the Assembly Screen for Cobionts and Contaminants (ASCC) pipeline (article in preparation). Flat files and maps used in curation were generated in TreeVal ( Pointon *et al.*, 2023). Manual curation was primarily conducted using PretextView ( Harry, 2022), with additional insights provided by JBrowse2 ( Diesh *et al.*, 2023) and HiGlass ( Kerpedjiev *et al.*, 2018). Scaffolds were visually inspected and corrected as described by Howe *et al*. (2021). Any identified contamination, missed joins, and mis-joins were corrected, and duplicate sequences were tagged and removed. The sex chromosome was identified by synteny and read coverage statistics. The entire process is documented at https://gitlab.com/wtsi-grit/rapid-curation (article in preparation).

### Evaluation of the final assembly

The final assembly was post-processed and evaluated with the three Nextflow ( Di Tommaso *et al.*, 2017) DSL2 pipelines “sanger-tol/readmapping” ( Surana *et al.*, 2023a), “sanger-tol/genomenote” ( Surana *et al.*, 2023b), and “sanger-tol/blobtoolkit” ( Muffato *et al.*, 2024). The pipeline sanger-tol/readmapping aligns the Hi-C reads with bwa-mem2 ( Vasimuddin *et al.*, 2019) and combines the alignment files with SAMtools ( Danecek *et al.*, 2021). The sanger-tol/genomenote pipeline transforms the Hi-C alignments into a contact map with BEDTools ( Quinlan & Hall, 2010) and the Cooler tool suite ( Abdennur & Mirny, 2020), which is then visualised with HiGlass ( Kerpedjiev *et al.*, 2018). It also provides statistics about the assembly with the NCBI datasets ( Sayers *et al.*, 2024) report, computes *k*-mer completeness and QV consensus quality values with FastK and MERQURY.FK, and a completeness assessment with BUSCO ( Manni *et al.*, 2021).

The sanger-tol/blobtoolkit pipeline is a Nextflow port of the previous Snakemake Blobtoolkit pipeline ( Challis *et al.*, 2020). It aligns the PacBio reads with SAMtools and minimap2 ( Li, 2018) and generates coverage tracks for regions of fixed size. In parallel, it queries the GoaT database ( Challis *et al.*, 2023) to identify all matching BUSCO lineages to run BUSCO ( Manni *et al.*, 2021). For the three domain-level BUSCO lineage, the pipeline aligns the BUSCO genes to the Uniprot Reference Proteomes database ( Bateman *et al.*, 2023) with DIAMOND ( Buchfink *et al.*, 2021) blastp. The genome is also split into chunks according to the density of the BUSCO genes from the closest taxonomically lineage, and each chunk is aligned to the Uniprot Reference Proteomes database with DIAMOND blastx. Genome sequences that have no hit are then chunked with seqtk and aligned to the NT database with blastn ( Altschul *et al.*, 1990). All those outputs are combined with the blobtools suite into a blobdir for visualisation.

The genome assembly and evaluation pipelines were developed using the nf-core tooling ( Ewels *et al.*, 2020), use MultiQC ( Ewels *et al.*, 2016), and make extensive use of the Conda package manager, the Bioconda initiative ( Grüning *et al.*, 2018), the Biocontainers infrastructure ( da Veiga Leprevost *et al.*, 2017), and the Docker ( Merkel, 2014) and Singularity ( Kurtzer *et al.*, 2017) containerisation solutions.

Table 4 contains a list of relevant software tool versions and sources.

**Table 4.** Software tools: versions and sources.

Software tool	Version	Source
BEDTools	2.30.0	https://github.com/arq5x/bedtools2
BLAST	2.14.0	ftp://ftp.ncbi.nlm.nih.gov/blast/executables/blast+/
BlobToolKit	4.3.7	https://github.com/blobtoolkit/blobtoolkit
BUSCO	5.4.3 and 5.5.0	https://gitlab.com/ezlab/busco
bwa-mem2	2.2.1	https://github.com/bwa-mem2/bwa-mem2
Cooler	0.8.11	https://github.com/open2c/cooler
DIAMOND	2.1.8	https://github.com/bbuchfink/diamond
fasta_windows	0.2.4	https://github.com/tolkit/fasta_windows
FastK	427104ea91c78c3b8b8b49f1a7d6bbeaa869ba1c	https://github.com/thegenemyers/FASTK
Gfastats	1.3.6	https://github.com/vgl-hub/gfastats
GoaT CLI	0.2.5	https://github.com/genomehubs/goat-cli
Hifiasm	0.19.5-r587	https://github.com/chhylp123/hifiasm
HiGlass	44086069ee7d4d3f6f3f0012569789ec138f42b84a a44357826c0b6753eb28de	https://github.com/higlass/higlass
Merqury.FK	d00d98157618f4e8d1a9190026b19b471055b22e	https://github.com/thegenemyers/MERQURY.FK
MitoHiFi	3	https://github.com/marcelauliano/MitoHiFi
MultiQC	1.14, 1.17, and 1.18	https://github.com/MultiQC/MultiQC
NCBI Datasets	15.12.0	https://github.com/ncbi/datasets
Nextflow	23.04.0-5857	https://github.com/nextflow-io/nextflow
PretextView	0.2	https://github.com/sanger-tol/PretextView
purge_dups	1.2.5	https://github.com/dfguan/purge_dups
samtools	1.16.1, 1.17, and 1.18	https://github.com/samtools/samtools
sanger-tol/ascc	-	https://github.com/sanger-tol/ascc
sanger-tol/ genomenote	1.1.1	https://github.com/sanger-tol/genomenote
sanger-tol/ readmapping	1.2.1	https://github.com/sanger-tol/readmapping
Seqtk	1.3	https://github.com/lh3/seqtk
Singularity	3.9.0	https://github.com/sylabs/singularity
TreeVal	1.0.0	https://github.com/sanger-tol/treeval
YaHS	1.2a.2	https://github.com/c-zhou/yahs

### Genome annotation

The Ensembl Genebuild annotation system ( Aken *et al.*, 2016) was used to generate annotation for the *Catocala sponsa* assembly (GCA_963564715.1) in Ensembl Rapid Release at the EBI. Annotation was created primarily through alignment of transcriptomic data to the genome, with gap filling via protein-to-genome alignments of a select set of proteins from UniProt ( UniProt Consortium, 2019).

### Wellcome Sanger Institute – Legal and Governance

The materials that have contributed to this genome note have been supplied by a Darwin Tree of Life Partner. The submission of materials by a Darwin Tree of Life Partner is subject to the **‘Darwin Tree of Life Project Sampling Code of Practice’**, which can be found in full on the Darwin Tree of Life website here. By agreeing with and signing up to the Sampling Code of Practice, the Darwin Tree of Life Partner agrees they will meet the legal and ethical requirements and standards set out within this document in respect of all samples acquired for, and supplied to, the Darwin Tree of Life Project.

Further, the Wellcome Sanger Institute employs a process whereby due diligence is carried out proportionate to the nature of the materials themselves, and the circumstances under which they have been/are to be collected and provided for use. The purpose of this is to address and mitigate any potential legal and/or ethical implications of receipt and use of the materials as part of the research project, and to ensure that in doing so we align with best practice wherever possible. The overarching areas of consideration are:

• Ethical review of provenance and sourcing of the material

• Legality of collection, transfer and use (national and international)

Each transfer of samples is further undertaken according to a Research Collaboration Agreement or Material Transfer Agreement entered into by the Darwin Tree of Life Partner, Genome Research Limited (operating as the Wellcome Sanger Institute), and in some circumstances other Darwin Tree of Life collaborators.

## Data Availability

European Nucleotide Archive: *Catocala sponsa*. Accession number PRJEB66398; https://identifiers.org/ena.embl/PRJEB66398 ( Wellcome Sanger Institute, 2024). The genome sequence is released openly for reuse. The *Catocala sponsa* genome sequencing initiative is part of the Darwin Tree of Life (DToL) project. All raw sequence data and the assembly have been deposited in INSDC databases. Raw data and assembly accession identifiers are reported in Table 1 and Table 2.

## Author information

Members of the Natural History Museum Genome Acquisition Lab are listed here: https://doi.org/10.5281/zenodo.12159242.

Members of the Darwin Tree of Life Barcoding collective are listed here: https://doi.org/10.5281/zenodo.12158331

Members of the Wellcome Sanger Institute Tree of Life Management, Samples and Laboratory team are listed here: https://doi.org/10.5281/zenodo.12162482.

Members of Wellcome Sanger Institute Scientific Operations: Sequencing Operations are listed here: https://doi.org/10.5281/zenodo.12165051.

Members of the Wellcome Sanger Institute Tree of Life Core Informatics team are listed here: https://doi.org/10.5281/zenodo.12160324.

Members of the Tree of Life Core Informatics collective are listed here: https://doi.org/10.5281/zenodo.12205391.

Members of the Darwin Tree of Life Consortium are listed here: https://doi.org/10.5281/zenodo.4783558.
