# Supplementary material for: The genome sequence of the Dark Crimson Underwing moth, Catocala sponsa Linnaeus, 1767
Source: Wellcome Open Res. 2024 Jul 26;9:412. [Version 1] doi: 10.12688/wellcomeopenres.22759.1 (PMC11417456; doi:10.12688/wellcomeopenres.22759.1)
Supplement: Peer review history

# Reviewer report: Reviewer response for version 1

**DOI:** 10.21956/wellcomeopenres.25063.r95841

**Published:** 2024-09-25

**Authors:** Sivasankaran Kuppusamy

**Affiliations:** 1 Entomology Research Institute, Loyola College, Chennai, India

Authors have done the whole genome sequence of *Catocala sponsa* Linnaeus, 1767. Authors have produced comprehensive data through the assembly. Through the assembly and annotations authors have received 803.70 Mb span of genome sequence, 32 chromosomes, 13, 493 protein-coding genes, 25,901 of gene transcripts and 2,838 non-coding genes. The work is appreciable and commendable. 

**Comments on manuscript:**

- Author’s name can be included for the species name. The first sentence can be modified as *Catocala sponsa* Linnaeus, 1767 Dark Crimson Underwing……….
- The authors have given the genus name *Catocala sponsa* somewhere complete form and someplace abbreviated form. The genus name may be given in full the first time, and then in abbreviated form later on, such as *C. sponsa.*

Above all, I confirm that the manuscript meets the necessary scientific standard and is suitable for indexing.

Are sufficient details of methods and materials provided to allow replication by others?

Yes

Is the rationale for creating the dataset(s) clearly described?

Yes

Are the datasets clearly presented in a useable and accessible format?

Yes

Are the protocols appropriate and is the work technically sound?

Yes

Reviewer Expertise:

Phylogenetic analysis of the superfamily Noctuoidea moths using mitochondrial genome sequence

I confirm that I have read this submission and believe that I have an appropriate level of expertise to confirm that it is of an acceptable scientific standard.

# Reviewer report: Reviewer response for version 1

**DOI:** 10.21956/wellcomeopenres.25063.r98549

**Published:** 2024-09-22

**Authors:** Paul Hickner

**Affiliations:** 1 Agricultural Research Service, United States Department of Agriculture, TX, USA

The authors present the nuclear and mitochondrial genome assemblies for *Catocala sponsa*, the Dark Crimson Underwing moth. Sequencing was conducted using the PacBio Revio platform to produce ~76X coverage of the genome with HIFI reads. Scaffolding of the 122 contigs was done using Hi-C with ~113X coverage. The assembly appears highly contiguous and complete. I believe this is a high quality genome assembly.

 I could not find details on the Hi-C library prep. Please make this available to the reader.

 It is difficult to assess the quality of the annotations. It was not clear if Busco was used to assess the annotations or if they were similar to other closely related taxa. 

 Also, in the gene annotation section, it should be stated (broadly) what “select set” of proteins were used for protein-to-genome alignments for gap filling. i.e. were they all Lepidopteran? 

 The justification/importance of this genome was not clearly stated.

Are sufficient details of methods and materials provided to allow replication by others?

Partly

Is the rationale for creating the dataset(s) clearly described?

No

Are the datasets clearly presented in a useable and accessible format?

Yes

Are the protocols appropriate and is the work technically sound?

Yes

Reviewer Expertise:

Insect genomics, behavior, gene family evolution

I confirm that I have read this submission and believe that I have an appropriate level of expertise to confirm that it is of an acceptable scientific standard.

# Reviewer report: Reviewer response for version 1

**DOI:** 10.21956/wellcomeopenres.25063.r98551

**Published:** 2024-09-12

**Authors:** Daniel Berner

**Affiliations:** 1 Department of Environmental Sciences, Zoology and Evolution, University of Basel, Basel, Switzerland

The author and the involved teams here present a genome assembly and annotation of the moth *Catocala sponsa*, based on a 2020 female specimen from the UK. The genome is assembled by combining HIFI PacBio reads (around 76x read depth) and HI-C chromosome conformation data. The genome is c. 804 Mb in length, and assembled to 30 autosomes plus the W and Z chromosomes and the mitochondrial chromosome. The genome exhibits excellent completeness (e.g. as assessed by BUSCO) and contiguity. The assembly is complemented by a gene annotation based on RNA sequencing of the specimen’s abdominal tissue and including some 13,5 k protein-coding genes.

**In my view, this genome report is well done and ready for approval. I just have a few very minor comments:**

 1) The Background passage starts by mentioning the status of this species in ‘New Forest’, which must be somewhere in the UK. However, I personally have no notion of this place, and was initially missing more general distribution information relevant to the world-wide readership. The Background section later gives more general indication, but the author could consider starting this section with general information on the species’ ecology and distribution, and only then move to the specific details relevant to the UK?

 2) The Assembly section mixes past and present tense. This is again not a crucial issue, but I would recommend to change all of this passage to past tense.

 3) The Assembly curation passage mentions that “The sex chromosome was identified by synteny …”. I proposed to change this to “The sex chromosomeS WERE identified...”, since this concerns both the W and Z, right?

Are sufficient details of methods and materials provided to allow replication by others?

Yes

Is the rationale for creating the dataset(s) clearly described?

Yes

Are the datasets clearly presented in a useable and accessible format?

Yes

Are the protocols appropriate and is the work technically sound?

Yes

Reviewer Expertise:

evolution; ecology; population genomics

I confirm that I have read this submission and believe that I have an appropriate level of expertise to confirm that it is of an acceptable scientific standard.
